# Anemochorous and zoochorous seeds of trees from the Brazilian savannas differ in fatty acid content and composition

**DOI:** 10.1093/aobpla/plad042

**Published:** 2023-08-17

**Authors:** Augusto Cesar Franco, Risolandia Bezerra de Melo, Cristiane Silva Ferreira, Thomas Christopher Rhys Williams

**Affiliations:** Department of Botany, University of Brasilia, Campus Universitário Darcy Ribeiro, Brasilia, DF 70910-900, Brazil; Department of Physiological Sciences, University of Brasilia, Campus Universitário Darcy Ribeiro, Brasilia, DF 70910-900, Brazil; Department of Botany, University of Brasilia, Campus Universitário Darcy Ribeiro, Brasilia, DF 70910-900, Brazil; Department of Botany, University of Brasilia, Campus Universitário Darcy Ribeiro, Brasilia, DF 70910-900, Brazil

**Keywords:** Cerrado, seed oil, seed reserves, seed storage lipids, seed traits, transesterification

## Abstract

Fatty acids (FAs) stored as triacylglycerols (TAGs) are an important source of carbon and energy for germination and seedling development, particularly for plants with small wind-dispersed seeds, allowing greater efficiency in storing both energy and carbon. These plants should be under strong selection to produce seeds rich in FAs and with large amounts of saturated FAs. Their closely packed single-chain configuration allows greater packing, more carbon and energy per unit mass, and are less costly to produce. Efficient carbon storage would be less crucial for zoochorous species, which can reach much larger seed sizes (mass). We analysed the transesterified FA profile from seeds of 22 anemochorous and zoochorous tree species from the Cerrado savannas of Central Brazil. We tested if seed FA content covaried with seed mass and if anemochorous and zoochorous seeds differed in FA contents and distribution. Fatty acids were an important seed source of carbon and energy for most species. Fifteen different FAs were identified. Oleic, linoleic and linolenic tended to be the predominant unsaturated FAs. Oleic acid corresponded to more than 60 % of the total transesterified FAs in seeds of *Kielmeyera coriacea, Qualea dichotoma* and *Triplaris americana*. Linoleic acid corresponded to more than 50 % of total FA in *Dalbergia miscolobium, Parkia platycephala* and *Ferdinandusa elliptica* while linolenic acid was the dominant component in *Inga cylindrica*. Across species, palmitic and stearic were the dominant saturated FAs. The only exception was lauric acid (68 % of total FA) in seeds of *Qualea grandiflora*. On a log_10_ scale, as the seed increased in mass, accumulation of FAs tends to proceed at a faster rate in anemochorous species than in zoochorous species. They also became increasingly richer in saturated FAs. Zoochorous species had seed TAGs with higher proportion of polyunsaturated FAs.

## Teaser

Fatty acids (FAs) stored as triacylglycerols (TAGs) in seeds are an important source of carbon and energy for germination and seedling development, particularly for plants with small wind-dispersed seeds, allowing greater efficiency in storing both energy and carbon. We analysed the FA profile from seeds of 22 wind- and animal-dispersed tree species from the Cerrado savannas of Brazil. Fifteen different FAs were identified. Oleic, linoleic and linolenic tended to be the predominant unsaturated FAs while palmitic and stearic were the dominant saturated FAs for most species. Wind-dispersed species produced seeds that not only were richer in FAs but in saturated FAs. Their closely packed carbon chains would allow greater carbon and energy per unit mass and more efficient carbon storage. Zoochorous species, which reached larger seed sizes, had seed TAGs with higher proportion of polyunsaturated FAs.

## Introduction

Seeds contain carbon and mineral nutrient reserves that are used to support embryo metabolism and seedling development (Quettier and Eastmond 2009; Bewley *et al.* 2013; De Melo *et al.* 2015). Seed carbon reserves can be compacted and stored as fatty acids (FAs) in the form of triacylglycerol (TAG), which has high energy and nutritional value (Graham 2008). In contrast to membrane lipids, the structure and physicochemical properties of seed storage lipids are less constrained, and many different FA structures have been described (Voelker and Kinney 2001; Matthäus 2012). The study of the composition of seed FAs is interesting not only for providing basic information for biochemical studies of the biosynthesis and catabolism of FAs which can potentially reveal novel insights into how biosynthetic pathways evolved in plants, but also for the understanding of the biochemical mechanisms that promote their biosynthesis (Broun *et al.* 1998; Vanhercke *et al.* 2011), their role in plant metabolism and development and their importance for the plant to successfully cope with abiotic and biotic stresses (He and Ding 2020; He *et al.* 2020). Seed storage lipids represent an important source of carbon and energy for germination and early seedling growth of many species in tropical regions such as rain forests (Finkelstein and Grubb 2002), deciduous forests (Soriano *et al.* 2011; Harand *et al.* 2016) and savannas (De Melo *et al.* 2020).

Early seedling development is dependent on the carbon reserves of the seed until the seedling is photosynthetically competent (Kitajima 2002; Kitajima and Myers 2008). The amount of carbon that can be stored in the seed and made available to the growing seedling is the result of seed carbon allocation into carbohydrates, proteins, and lipids and on seed size, both of which are associated with mode of dispersal (Finkelstein and Grubb 2002; De Melo *et al.* 2020). Animal-dispersed seeds are usually heavier than 100 mg, while most wind-dispersed seeds range in mass from 0.01 to 200 mg (Leishman *et al*. 2000; De Melo *et al.* 2020). Thus, allocation to compacted carbon reserves should be more relevant for the small wind-dispersed seeds, allowing greater efficiency in the use of the seed space for storing both energy and carbon units, despite the higher energy cost incurred for accumulation of assimilate in the form of FAs (Borisjuk *et al.* 2013). Zoochorous seeds tend to be less constrained in patterns of carbon allocation to storage. Depending on the species, starch, lipids or soluble sugars are the predominant seed carbon reserve (De Melo *et al.* 2020).

Since the primary function of TAGs is to store energy and carbon for the seed, we might expect wind-dispersed seeds to be under strong selection to have higher proportions of saturated FAs in seed TAGs, which have a higher melting point than unsaturated ones (Linder 2000). Their single-chain configuration can attain a lower energy configuration, with straight closely packed chains, unlike the unsaturated FAs. The tight packing allows seeds to store more chemical energy and carbon per unit mass. Conversely, we would not expect zoochorous seeds to be as constrained in terms of thermal stability and carbon storage efficiency and could afford higher proportions of monounsaturated and polyunsaturated FAs that are more fluid. On a broad geographical scale, seeds of tropical species tend to be richer in oils and to form TAGs with a higher proportion (>15 %) of saturated FAs than seeds of species from temperate zones, which are subjected to cooler temperatures during germination (Linder 2000; Sanyal *et al.* 2018). However, we are not aware of comparative studies of seed FA composition and contents in anemochorous and zoochorous seeds.

Here we focus on transesterifiable FA composition and contents in anemochorous and zoochorous seeds of trees of the Cerrado of Central Brazil. The Cerrado is a tropical savanna biome that originally stretched across two million square kilometres. A variety of seeds of native species serves as food for the local and visiting fauna (Kuhlmann and Ribeiro 2016; Darosci *et al.* 2017). Seeds of several native Cerrado trees are consumed by the local human population (Fernandes *et al.* 2010; Nunes *et al.* 2014; Alves *et al.* 2016; Silva and Fonseca 2016), or used for extraction of oils for cooking or herbal medicine (Silva and Fonseca 2016; Araújo *et al.* 2018). However, there is limited information on the composition of FAs in seeds of Cerrado tree species (Mayworm and Salatino 2002; Alves *et al.* 2016; Silva and Fonseca 2016; Cornelio-Santiago *et al.* 2021). The region is also undergoing extensive deforestation (Franco *et al.* 2014; Spera *et al.* 2016), meaning that systematic collection and analysis of seed oils from yet uncharacterized species of a large range of plant families is even more crucial.

Anemochory and zoochory are the predominant dispersal modes in Cerrado trees and with a high potential for long-distance dispersal (Kuhlmann and Ribeiro 2016). Seeds of anemochorous species are generally dispersed at the end of the dry season and dry-to-rainy transition while zoochorous species as a group do not show a seasonal pattern of fruit production and seed dispersal (Escobar *et al.* 2018). We expected that anemochorous seeds would be richer in FAs and saturated FAs as a compensatory mechanism because they would provide more energy and carbon units per unit mass and energetically are less costly to produce (He *et al.* 2020). This efficient form of storing carbon and energy would be more advantageous for allowing dispersal at long distance for small wind-dispersed seeds, besides providing sufficient carbon resources during early seedling establishment (Finkelstein and Grubb 2002). Conversely, as the seed increases in size, efficient carbon storage in FAs would be less crucial. To assess this, we analysed the transesterified FA profile from seeds of 22 Cerrado tree species distributed across 10 plant families and tested if seed FA contents and distribution covaried with seed size (mass).

## Material and Methods

Seeds were collected from savannas and gallery forests throughout the Federal District, which is located in the centre of the Brazilian Cerrado, being delimited by the parallels 15°30’ and 16°03’ S and meridians 47°25’ and 48°12’ W. Although savanna is the predominant type of vegetation in the region, gallery forests are common along streams. Mature fruits were collected from 3 to 10 individuals of each species, with individuals of a given species at least 50 m apart. A total of 22 tree species were sampled from August 2016 to August 2017. Species names and families follow the Brazilian Flora species list (Flora e Funga do Brasil; http://floradobrasil.jbrj.gov.br/; accessed on 19 April 2021). The region is characterized by strong rainfall seasonality with a well-defined winter drought period (Franco 2002). Intense and rapid surface fires are common in the dry period (Hoffmann *et al.* 2009). Seed dispersal of anemochorous species was concentrated in the dry period (June to September) or in the dry-to-rainy transition (October) **[see****Supporting Information—Table S1****]**. The only exception was *Vochysia pyramidalis* which dispersed seeds in the late wet season (March–April). Peak fruiting for zoochorous species was in the late dry season and during the dry-to-rainy transition. However, several species produced fruits throughout the rainy season (November to April) and during the rainy-to-dry transition (May).

**Figure 1.**
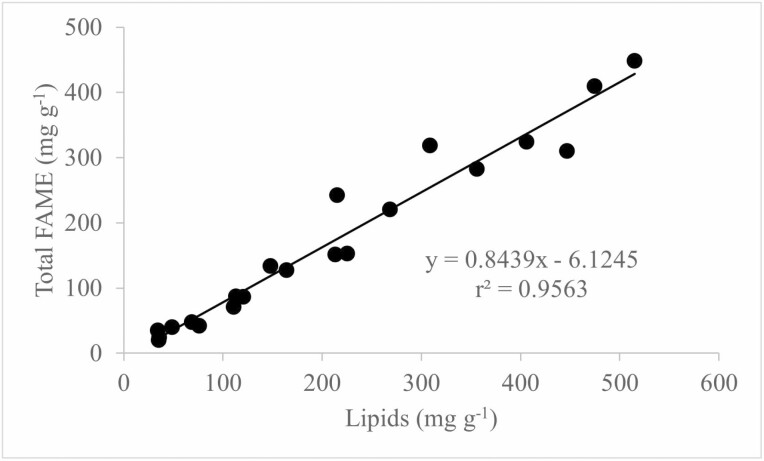
Relationship between lipids and fatty acid methyl esters for seeds of tree species from the Cerrado of Central Brazil. Ordinary least square regression was used to fit the data. Data on lipids were from De Melo *et al.* (2020). Data expressed on a seed dry mass basis. Each data point is from a different species.

Prior to FAs analysis, hand-picked, mature seeds of each species were mixed in a single lot to obtain pooled samples. After manual removal of the seed coat, seeds were lyophilized until a constant mass was reached (minimum of 72 h) and subsequently ground to powder in a knife mill (MDR302, Di Grano) followed by storage at −20 °C until processed. Fatty acids in dry biomass were converted to their methyl esters (FAMES) using a hydrochloric acid/methanol *in situ* method adapted from Laurens et al. (2012). Briefly, 10 mg of dry biomass was incubated with 0.2 mL 2:1 chloroform:methanol (v/v), 0.3 mL HCl in methanol (5%, v/v) and 25 µg methyl tridecanoate as an internal standard in a sealed glass vial at 85 °C for 1 h. After cooling to room temperature, 1 mL hexane was added, samples were shaken gently for 1 h and the upper hexane phase that contained FAMEs recovered for analysis. Transesterification was performed on triplicate biomass samples. Samples were analysed on a GC-MS (Agilent, 7890-5975) equipped with a 30-m INNOWAX (Agilent Technologies) capillary column. 1 μL of sample was injected with an injector temperature of 250 °C and split ratio of 30:1. Separation was achieved using 1 mL mL^−1^ constant helium gas flow and a temperature program starting at 70 °C, ramping to 130 °C at 20 °C min^−1^ and then to 260 °C at 10 °C min^−1^, before maintaining the final temperature for 5 min. Electron impact ionization was performed at −70 eV and mass spectra were acquired in scan mode. FAMES were identified by comparison of mass spectra with the NIST mass spectral database using the MSSearch Software and by comparison of retention times with authentic standards (Supelco 37 FAME mixture, Sigma-Aldrich, USA). Quantification was carried out using the Agilent ChemStation software and standard curves were constructed using a mixture of FAMES (Supelco 37 FAME mixture, Sigma-Aldrich, USA). The method was validated by comparison of total FAME on a dry mass basis with a previous gravimetric analysis of lipids from seeds of the same species (De Melo *et al.* 2020; Fig. 1), which demonstrated good agreement between the two methods over a wide range of total FAME concentrations. Overall, total FAME corresponded to about 85% of total lipids in the seeds.

## Statistical Analyses

We used standardized major axis (SMA) for the relationships between seed mass and FA contents and between relative proportions of oleic acid and linoleic + linolenic acids (Warton *et al.* 2006). We applied the likelihood ratio test to test for differences in slopes and the Wald test for differences in intercepts between anemochorous and zoochorous species and for shifts along the common axis when no differences in slopes and intercepts were detected. We also tested if the slopes of the fitted lines differed from 1. These analyses were performed with the SMATR freeware program, version 2.0 (Warton *et al.* 2006).

## Results

### Seed transesterified fatty acid composition for the 22 studied tree species

Transesterified FAs comprised more than 10% of the seed dry mass in 12 out of the 22 species analysed (Table 1). The average proportion of transesterified FAs was 16.35% ranging from 2.02% in seeds of *Triplaris americana* to 44.80% in seeds of *Kielmeyera speciosa*. *Handroanthus impetiginosus, Kielmeyera coriacea, Qualea grandiflora* and *Dipteryx alata* also had high amounts of FAs in the seeds, over 30% of seed dry mass. *Triplaris americana* was the only species that had a proportion of saturated FAs <15.0% while *Brosimum gaudichaudii* and *Inga cylindrica* were the only species that did not have any monounsaturated FA in the seed (Table 1).

**Table 1.** Seed dry mass, fatty acid concentration (as % of seed dry mass), fatty acid content and contribution (as % of total FA) of saturated (Sat), monounsaturated (Monounsat) and polyunsaturated (Polyunsat) fatty acids in seeds of 22 Cerrado tree species. Seed dry mass from De Melo *et al.* (2020). NA: not available.

Species	Family	Seed mass (mg)	FA (%)	FA (mg)	Sat (%)	Monounsat (%)	Polyunsat (%)
Anemochorous							
*Dalbergia miscolobium* Benth.	Fabaceae	68.53	8.70	5.96	29.00	10.88	60.12
*Ferdinandusa elliptica* (Pohl) Pohl	Rubiaceae	5.62	22.05	1.24	27.32	20.10	52.58
*Handroanthus impetiginosus* (Mart. ex DC.) Mattos	Bignoniaceae	52.20	32.41	16.92	40.35	31.80	27.86
*Kielmeyera coriacea* Mart.	Calophyllaceae	111.03	40.98	45.50	34.88	62.19	2.92
*Kielmeyera speciosa* A. St.-Hill	Calophyllaceae	123.15	44.80	55.17	39.31	15.27	45.43
*Myracrodruon urundeuva* Allemão	Anacardiaceae	17.78	15.29	2.72	36.96	23.41	39.62
*Plathymenia reticulata* Benth.	Fabaceae	54.47	24.23	13.20	34.48	52.86	12.66
*Qualea dichotoma* (Mart.) Warm. ex Wille	Vochysiaceae	10.18	13.36	1.36	19.83	77.91	2.26
*Qualea grandiflora* Mart.	Vochysiaceae	139.32	31.00	43.19	85.81	10.15	4.04
*Tabebuia aurea* (Silva Manso) Benth.	Bignoniaceae	178.93	15.16	27.13	37.78	50.18	12.04
*Triplaris americana* L.	Polygonaceae	32.27	2.02	0.65	4.38	77.73	17.89
*Vochysia pyramidali*s Mart.	Vochysiaceae	20.01	28.23	5.65	47.66	50.05	2.29
Zoochorous							
*Blepharocalyx salicifolius* (Kunth) O. Berg	Myrtaceae	20.26	7.11	1.44	49.00	21.77	29.23
*Brosimum gaudichaudii* Trécul	Moraceae	601.75	2.41	14.50	27.02	0.00	72.98
*Dipteryx alata* Vogel	Fabaceae	1308.97	31.82	416.51	18.38	60.30	21.33
*Enterolobium contortisiliquum* (Vell.) Morong	Fabaceae	755.17	3.48	26.28	25.23	31.20	43.57
*Inga cylindrica* (Vell.) Mart.	Fabaceae	531.21	3.96	21.04	15.42	0.00	84.58
*Myrcia fenzliana* O.Berg	Myrtaceae	31.10	4.75	1.48	39.70	25.54	34.81
*Ormosia arborea* (Vell.) Harms.	Fabaceae	546.10	4.23	23.10	16.06	54.23	29.71
*Parkia platycephala Benth.*	Fabaceae	75.39	12.71	9.58	15.70	9.72	74.57
*Salacia crassifolia* (Mart. Ex Schult.) G. Don	Celastraceae	NA	2.48	NA	45.20	23.21	31.59
*Stryphnodendron adstringens* (Mart.) Coville	Fabaceae	69.43	8.61	5.98	23.37	39.33	37.30

The unsaturated FAs oleic (C18:1^Δ9^, OA) and/or linoleic (C18:2^Δ9,12^, LA) acids were amongst the dominant components in most species (Table 2). LA was present in the oil composition of seeds of all species, while OA was present in the seed oil of 20 species. OA corresponded to more than 60% of the total transesterified FAs in seeds of *K. coriacea, Qualea dichotoma* and *T. americana* while LA corresponded to more than 50 % of total FA in *Dalbergia miscolobium, Ferdinandusa elliptica* and *Parkia platycephala.* Linolenic (C18:3) was the dominant component in seeds of *I. cylindrica*. It was also found in relatively high amounts in seeds of *B. gaudichaudii*. Among the saturated FAs, palmitic acid (C16:0) was widely distributed, being a seed oil component in all species (4–43 %). Stearic acid (C18:0) was present in seed oils of 19 species, comprising <1 % (*Q. grandiflora*) to 33.5 % (*V. pyramidalis*) of the total transesterified FAs.

**Table 2.** Fatty acid composition in seeds of 22 Cerrado tree species. Data expressed as percentages of the total fatty acid concentration. Values of 0 indicate that the corresponding FAME was not detected whilst <1.0 indicates that the FAME was below the threshold for quantification. Fatty acids: capric (C10:0), lauric (C12:0), myristic (C14:0), palmitic (C16:0), palmitoleic (C16:1), stearic (C18:0), oleic (C18:1), linoleic (C18:2), linolenic (C18:3), arachidic (C20:0), paullinic (C20:1), eicosadienoic (C20:2), behenic (C22:0), erucic (C22:1), lignoceric (C24:0).

Species	C10:0	C12:0	C14:0	C16:0	C16:1	C18:0	C18:1	C18:2	C18:3	C20:0	C20:1	C20:2	C22:0	C22:1	C24:0
Anemochorous															
*D. miscolobium*	0	0	0	17.52	0	6.98	10.88	51.45	8.67	<1.0	0	0	3.35	0	<1.0
*F. elliptica*	0	0	0	13.98	0	12.86	20.04	52.29	0	<1.0	<1.0	<1.0	0	0	0
*H. impetiginosus*	0	0	0	23.24	0	15.14	31.08	14.38	13.48	1.82	<1.0	0	<1.0	0	0
*K. coriacea*	0	0	0	27.64	<1.0	6.08	61.93	2.71	<1.0	<1.0	<1.0	0	<1.0	0	0
*K. speciosa*	0	0	0	22.69	0	16.52	15.27	45.28	<1.0	<1.0	0	0	<1.0	0	0
*M. urundeuva*	0	0	0	23.49	0	13.37	23.41	37.56	2.06	<1.0	0	0	0	0	0
*P. reticulata*	0	0	0	25.72	<1.0	7.40	52.48	12.52	<1.0	<1.0	<1.0	0	<1.0	0	<1.0
*Q. dichotoma*	0	0	1.59	16.61	0	1.63	77.91	2.26	0	0	0	0	0	0	0
*Q. grandiflora*	4.64	68.28	5.20	7.23	0	<1.0	10.15	4.03	<1.0	0	0	0	0	0	0
*T. aurea*	0	0	0	21.15	<1.0	14.34	47.63	10.83	1.21	2.29	1.74	0	0	0	0
*T. americana*	0	0	0	4.38	0	0	77.73	17.49	<1.0	0	0	0	0	0	0
*V. pyramidali*s	0	0	0	7.71	0	33.50	47.02	2.29	0	6.26	3.02	0	<1.0	0	0
Zoochorous															
*B. salicifolius*	0	0	0	35.53	0	13.40	21.77	28.14	1.09	<1.0	0	0	0	0	0
*B. gaudichaudii*	0	0	0	27.02	0	0	0	44.56	28.42	0	0	0	0	0	0
*D. alata*	0	0	0	8.28	0	3.96	57.16	21.33	0	<1.0	3.11	0	3.33	<1.0	2.37
*E. contortisiliquum*	0	0	0	18.41	0	5.64	31.20	43.52	<1.0	1.18	0	0	0	0	0
*I. cylindrica*	0	0	0	15.42	0	0	0	37.57	47.01	0	0	0	0	0	0
*M. fenzliana*	0	<1.0	0	27.89	0	11.58	25.54	30.82	3.99	0	0	0	0	0	0
*O. arborea*	0	0	0	13.15	0	2.90	54.23	29.71	0	0	0	0	0	0	0
*P. platycephala*	0	0	0	7.97	0	7.74	9.72	69.71	4.86	0	0	0	0	0	0
*S. crassifolia*	0	0	0	42.89	0	2.31	23.21	30.45	1.14	0	0	0	0	0	0
*S. adstringens*	0	0	0	16.07	1.67	5.09	37.54	37.30	0	<1.0	<1.0	0	1.52	0	0

Among the 15 different FAs identified in the composition of the seed oils, six were of restricted distribution (Table 2). The rarest were capric (C10:0), eicosadienoic (C20:2) and erucic acids (C22:1). They were found in only one species each (*Q. grandiflora, F. elliptica* and *D. alata*, respectively). Myristic acid (C14:0) was only found in seeds of the two *Qualea* species (*Q. dichotoma* and *Q. grandiflora*). The saturated FA lauric acid (C12:0) was the dominant component (68%) in seeds of *Q. grandiflora,* the only species with a seed oil composed mostly of saturated FAs. Apart from *Q. grandiflora*, seeds of *Myrcia fenzliana* also had lauric acid in its composition, however in this case less than 1 % of the oil was composed of lauric acid. Lignoceric acid (C24:0) was present in seed oils of three species of Fabaceae, *D. miscolobium, D. alata* and *Plathymenia reticulata* (Table 2).

### Mode of dispersal and fatty acid content and composition

**Figure 2.**
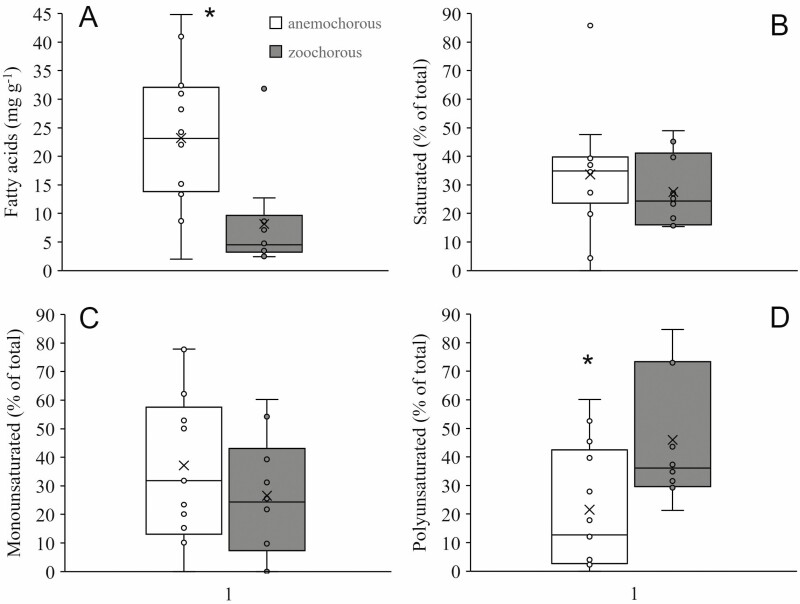
Transesterified fatty acid concentration (mg g^−1^ of seed dry mass) and the relative proportion of saturated, monounsaturated and polyunsaturated fatty acids in seeds of 22 anemochorous and zoochorous tree species from the Cerrado of Central Brazil. Asterisks denote significant differences (*P* < 0.05) by t tests after logit transformation of the data.

Twelve of the 22 species studied here had anemochorous seeds and 10 had zoochorous seeds (Table 1). Seed dry mass ranged from 5.62 to 178.93 mg for anemochorous seeds and from 20.26 to 1308.97 mg for zoochorous seeds, while the FA content in a seed ranged from 0.65 to 55.17 mg for anemochorous seeds and from 1.44 to 416.51 mg for zoochorous seeds. In comparison to zoochorous seeds, anemochorous seeds had higher concentrations of FAs (Fig. 2A). The two groups did not differ in the relative proportion of saturated and monounsaturated FAs (Fig. 2B and C), however, zoochorous seeds had higher relative proportions of polyunsaturated FAs (Fig. 2D).

**Figure 3.**
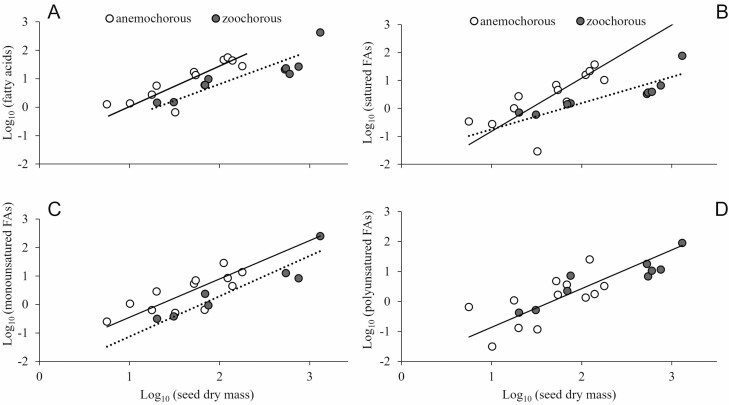
Allometric relationships between seed dry mass and total (A), saturated (B), monounsaturated (C), polyunsaturated (D) fatty acids contents in seeds of Cerrado tree species. Open circles represent anemochorous and full circles zoochorous species. Relationships were obtained with log_10_ transformed values. In (A) the scaling exponents did not differ statistically between the two groups (LRT = 0.885, *P* = 0.347), but the elevations were statistically different (Wald Statistic = 9.603, *P* = 0.002). Equation for anemochorous species: *Y* = −1.386 + 1.398*x, r*^2^ = 0.714, *P* = 0.001; for zoochorous species: *Y* = −1.432 + 1.102*x, r*^2^ = 0.788, *P* = 0.001. In (B) the scaling exponents differed statistically between the two groups (LRT = 4.950, *P* = 0.015). Equation for anemochorous species: *Y* = −2.727 + 1.9055*x, r*^2^ = 0.536, *P* = 0.007; for zoochorous species: *Y* = −1.692 + 0.9416*x, r*^2^ = 0.737, *P* = 0.003. In (C) the scaling exponents did not differ statistically between the two groups (LRT = 0.033, *P* = 0.854), but the elevations were statistically different (Wald Statistic = 7.362, *P* = 0.007). Equation for anemochorous species: *Y* = −1.803 + 1.352*x, r*^2^ = 0.590, *P* = 0.004; for zoochorous species: *Y* = −2.546 + 1.420*x, r*^2^ = 0.866, *P* = 0.002. In (D) the scaling exponents and the elevations did not differ statistically between the two groups (LRT = 2.02, *P* = 0.160 for scaling exponents; Wald statistic = 0.205, *P*= 0.651 for elevations) but with significant shift along common slope (Wald statistic = 5.194, *P* = 0.023). Equation: *Y* = −2.157 + 1.292*x, r*^2^ = 0.670, *P* < 0.001.

On a log_10_ scale, as seed mass increased the total FA content and the contents of saturated, monounsaturated and polyunsaturated FAs increased in seeds of anemochorous and zoochorous species (Fig. 3). The slopes of fitted lines between seed mass and total FA content were close to 1 (*F* = 4.065, *P* = 0.071 for anemochorous and *F* = 0.310, *P* = 0.595 for zoochorous species) and did not differ statistically between the two groups, but their elevations were different (Fig. 3A). As a result, for a given seed mass, seeds of anemochorous species had a larger amount of FAs. The contents of saturated FAs in seeds of anemochorous species increased exponentially (slope greater than 1 for the fitted line; *F* = 10.264, *P* = 0.009) as the seed increased in mass, while it increased linearly (slope not different from 1; *F* = 0.097, *P* = 0.765) for seeds of zoochorous species (Fig. 3B). *Brosimum gaudichaudii* and *I. cylindrica* did not have any monounsaturated FA in the seed (Table 1) and were not included in the analysis of relationship between seed mass and monounsaturated FAs. For the remaining species, the slopes of the fitted lines between seed mass and the contents of monounsaturated FAs were close to 1 (*F* = 2.291, *P* = 0.161 for anemochorous and *F* = 4.775, *P* = 0.081 for zoochorous species) and did not differ statistically between the two groups (Fig. 3C). Because their elevations differed, seeds of anemochorous species had greater amounts of monounsaturated FAs for a given seed mass. Anemochorous and zoochorous species shared a common axis of variation for the relationship between seed mass and the contents of polyunsaturated FAs (Fig. 3D). However, there was a significant shift along the common slope. Across species, seeds of zoochorous species reached larger mass that contained higher amounts of polysaturated FAs than anemochorous species. The slope of the fitted line did not differ from 1 (*F* = 3.861, *P* = 0.064).

**Figure 4.**
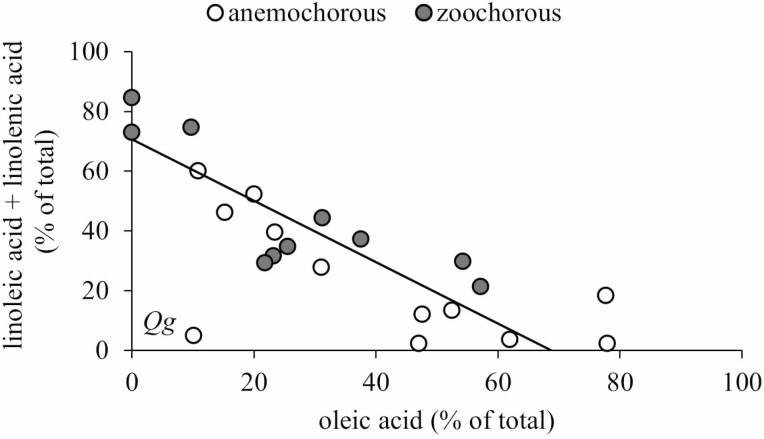
Relationship between the relative proportions of oleic acid and linoleic + linolenic acids in seeds of 22 anemochorous and zoochorous tree species from the Cerrado of Central Brazil. Open circles represent anemochorous and full circles zoochorous species. The scaling exponents and the elevations did not differ statistically between the two groups (LRT = 0.866, *P* = 0.354 for scaling exponents; Wald statistic = 0.607, *P*= 0.436 for elevations) and there was no significant shift along common slope (Wald statistic = 3.594, *P* = 0.058). The SMA regression equation: *Y* = 70.73 − 1.029*x*; *r*^2^= 0.744; and *P* < 0.001 without including *Qualea grandiflora* (Qg).

Across anemochorous and zoochorous species, oleic, linoleic and linolenic tended to be the predominant unsaturated FAs corresponding to 43–96 % of the FAs in the seeds (Table 2). Despite large variations in the proportion of these three FAs in the seeds, a tight negative correlation was found between the relative proportions of oleic acid and linoleic + linolenic acids (Fig. 4). The relationship was similar for anemochorous and zoochorous species and the slope of the fitted line did not differ from 1. The only species that did not fit this pattern was *Q. grandiflora*. This anemochorous species had seed oil composed mostly of saturated FAs.

## Discussion

Seeds of Cerrado tree species showed great variation in FA contents ranging from ~2 % to 45 % of seed dry mass and can reach values as high as 64 % in *Caryocar coriaceum* (Dresen *et al.* 1989) and 69 % in *Virola sebifera* (Da Rocha Filho *et al.* 1992), two tree species found in the Cerrado. They also synthetized a range of different FAs. In addition to the 15 different types of FAs recorded here, low levels (1 % or less) of several other FAs have been reported for Cerrado trees, for example, heptadecanoic (17:0), heptadecenoic (C17:1) and arachdonic (C20:4) acids in seeds of *Campomanesia xanthocarpa* (Santos *et al.* 2012), heptadecanoic acid in seeds of *Annona crassiflora* (Luzia and Jorge 2013), and docosahexaenoic acid in seeds of *Caryocar brasiliense* (De Lima *et al.* 2007). High levels of myristic acid have been reported in seeds of *V. sebifera* (Da Rocha Filho *et al.* 1992) and of cyclopropenoid FAs in seeds of *Sterculia striata* (Mangas *et al.* 2012). Large differences in content and composition were found between species of the same genus *Q. dichotoma* and *Q. grandiflora*. At species level, however, the types and contents of dominant FAs in the seeds were similar to those reported by other authors (Mayworm and Salatino 2002), indicating that these routes are conserved in these two species, regardless of the locality of seed collection. This strong genetic control over the biosynthetic pattern of FAs has been reported in other studies (e.g. Graham *et al.* 2016).

Oleic, linoleic and linolenic tended to be the predominant unsaturated FAs corresponding to 43 % to 96 % of the FAs in the seeds while palmitic and stearic were the most widely distributed saturated FAs. Seeds of anemochorous and zoochorous species shared the same axis of variation between the relative proportions of oleic acid and linoleic + linolenic acids (Fig. 4). The strong negative correlation between the relative proportions of oleic acid and linoleic + linolenic acids is not unexpected. The biosynthetic routes of these three C18 unsaturated FAs are coupled by a series of FA desaturases (Nayeri and Yarizade 2014).

Climatic conditions, such as ambient temperature, can influence the composition of FAs in seeds (Dornbos and Mullen 1992; Zhang *et al.* 2015), while the proportion of saturated and unsaturated FAs that make up the TAG affects its melting point. Relative to seeds of species from temperate zones, seeds of tropical species tend to form TAGs with a higher proportion (>15 %) of saturated FAs, which have a higher melting point than unsaturated ones (Linder 2000). Their single-chain configuration can attain a lower energy configuration, with straight closely packed chains, unlike the unsaturated FAs. Thus, the presence of higher contents or different types of saturated FAs could provide greater stability for cell membranes and organelles in species of warmer climates. In the present study, 77 % of the analysed species had saturated FA contents above 20 % of the total oil content of the seeds and only one (*Triplaris americana*) out of 22 species had <15 % of the seed oil content as saturated FAs. Both anemochorous and zoochorous Cerrado trees can have seed oils enriched in short chain saturated FAs. Lauric acid is the predominant FA in seeds of *Q. grandiflora*, an anemochorous species, and one of the most widespread tree species in the Cerrado region (Ratter *et al.* 2003). It is also the predominant FA in seeds of *Apeiba tibourbou*, a zoochorous tree (Martins and Conceição 2015). Palmitic acid is the predominant FA in seeds of several zoochorous species, that is, *C. brasiliense* (De Lima *et al*. 2007), *C. coriaceum* (Dresen *et al.* 1989), *S. crassifolia* (Table 2), *Dimorphandra mollis* (Mayworm and Salatino 1996), *Pachira retusa* (Pereira *et al.* 2013) and in *Eriotheca gracilipes* (Mayworm and Salatino 1996) an anemochorous species.

The Cerrado is subjected to strong rainfall seasonality, with well‐marked dry and wet seasons (Franco 2002). Seeds of anemochorous woody species are generally dispersed during the dry season and remain on the soil surface or slightly buried for weeks, under high temperatures, before germinating at the start of the rainy season (Salazar *et al.* 2011; Escobar *et al.* 2018). Although zoochorous species are not as constrained in terms of dispersal period, seeds dispersed in the rainy-to-dry season transition generally remain dormant and only germinate in the subsequent rainy season (Escobar *et al.* 2018). Optimum temperature for germination of Cerrado trees is between 25 and 30 °C (Brancalion *et al.* 2007; Ribeiro and Borghetti 2014); however, many species can germinate at higher temperatures albeit at lower rates (Ribeiro and Borghetti 2014) and seeds of many species can survive heat shock of short durations at temperatures resembling surface fires that typically occur in Cerrado savannas (Ribeiro *et al.* 2015). This suggests that thermal stability and thermal requirements for germination are similar for both types of seeds.

The total amount of seed carbon reserves as FAs and their composition can be, however, important to fuel growth and deeper extension of the root system in strongly seasonal savanna environments, enhancing seedling survival in the subsequent dry period (Groom and Lamont 2010; Lamont and Groom 2013). Despite being constrained by the amount of carbon they can store in their smaller sized seeds, anemochorous species can at least in part overcome this limitation by producing seeds that not only are richer in FAs but in saturated FAs (Fig. 3A and B). Their closely packed carbon chains would allow greater carbon and energy per unit mass and more efficient carbon storage. Across species, as the seed increased in mass, accumulation of FAs tends to proceed at a faster rate in anemochorous species than in zoochorous species. Seeds of anemochorous species also became increasingly richer in saturated FAs. In contrast, as seeds of zoochorous species increased in mass, the total contents of FAs and of saturated, mono and polyunsaturated FAs increased approximately linearly. Most zoochorous species had low concentrations of FAs (Table 1) and on average, zoochorous species had seed storage FAs with a higher proportion of polyunsaturated FAs than anemochorous species (Fig. 2). This suggests that seed TAGs in anemochorous and zoochorous seeds are subjected to different selective pressures or physiochemical constraints in terms of accumulation patterns and composition. Our focus here, however, was on seed TAGs as compact carbon storage compounds and their scaling relationships with seed mass. Other roles of seed FAs (Walley *et al.* 2013; He and Ding 2020; McInnes *et al.* 2023) were beyond the scope of our study.

The Cerrado has a diversity of species with different dispersion strategies that are reflected in a diversity of shapes, sizes and contents of reserves in the seeds (De Melo *et al.* 2020). Even though further studies are necessary to better address the role of seed FAs in germination and seedling establishment of tree species in these seasonally dry tropical ecosystems, we provide evidence that FAs are important sources of carbon and energy for seeds of anemochorous species, more specifically saturated FAs with their closed packed carbon chains. Zoochorous species are apparently less constrained in resource allocation to seed TAGs and tend to invest proportionally more in polyunsaturated FAs. In addition, knowing the composition of FAs as storage compound in seeds of native Cerrado tree species is important to add value to biodiversity, especially in a strongly threatened ecosystem such as the Cerrado, in which many species are in danger of disappearing due to increasing changes in land use. For some of the listed species, this is the first study on the content and type of FAs that are accumulated by their seeds.

## Sources of Funding

This work was supported by the Brazilian agencies Conselho Nacional de Desenvolvimento Científico e Tecnológico (CNPq; grant nos. 311362/2019-2 awarded to A.C.F. and 305288/2020- 2 awarded to T.C.R.W.), Coordenação de Aperfeiçoamento de Pessoal de Nível Superior (CAPES; finance code 001), Brazilian Innovation Agency (FINEP; CT-INFRA/FINEP/MCTIC) and Fundação de Amparo a Pesquisa do Distrito Federal (FAPDF; grant no. 00193-00000052/2019-48).

## Contributions by the Authors

A.C.F. and R.B.M. conceived and designed the study. R.B.M., C.S.F. and T.C.R.W. collected and analyzed the data. A.C.F., C.S.F. and T.C.R.W. analyzed and interpreted the results. A.C.F and C.S.F. wrote the first draft of the manuscript with the help of T.C.R.W. All authors gave final approval for publication.

## Supporting Information

The following additional information is available in the online version of this article –

**Table S1**: Information board with the period (months) of fruit collection. Data obtained from the literature (Ribeiro *et al.* 2018) or by consulting the material deposited in the Reflora - Virtual Herbarium. Available in: http://reflora.jbrj.gov.br/reflora/herbarioVirtual/. Accessed on 23 April 2023.

plad042_suppl_Supplementary_Table_S1

## Data Availability

The data that support the findings of this study are available from the corresponding author upon reasonable request

## Notes

**Form and Function. Chief Editor:** Kate McCulloh
