# Supplementary material for: Anemochorous and zoochorous seeds of trees from the Brazilian savannas differ in fatty acid content and composition
Source: AoB Plants. 2023 Aug 17;15(4):plad042. doi: 10.1093/aobpla/plad042 (PMC10433789; doi:10.1093/aobpla/plad042)
Supplement: plad042_suppl_Supplementary_Table_S1 [file plad042_suppl_supplementary_table_s1.pdf]

Supporting Information Table 1: Information board with the period (months) of fruit collection. Data obtained from the literature (Ribeiro *et al.* 2018) or by consulting the material deposited in the Reflora - Virtual Herbarium. Available in: http://reflora.jbrj.gov.br/reflora/herbarioVirtual/. Accessed on 23 April 2023.

**Dispersion**	**Species**	**Fruit harvest period (month)**											
		Jan	Feb	Mar	Apr	May	Jun	Jul	Aug	Sep	Oct	Nov	Dec
**Anemochorous**	*Dalbergia miscolobium*[1]					■	■	■	■	■	■		
	Ferdinandusa elliptica[2]								■	■	■		
	Handroanthus impetiginosus[1]										■	■	
	Kielmeyera coriacea[1]									■	■	■	.
	Kielmeyera speciosa[1]							■	■	■			
	Myracrodruon urundeuva[1]								■	■	■		
	Plathymenia reticulata[1]											■	■
	Qualea dichotoma[1]	■	■						■	■	■	■	■
	Qualea grandiflora[1]								■	■			
	Tabebuia aurea[1]									■	■		
	Triplaris americana[1]	■										■	■
	Vochysia pyramidalis[2]			■	■								
**Zoochorous**	*Brosimum gaudichaudii*[1]	■								■	■	■	■
	Blepharocalyx salicifolius[1]	■	■	■	■								■
	Dipteryx alata[1]								■	■	■	■	
	Enterolobium contortisiliquum[1]								■	■	■	■	
	Inga cylindrica[1]									■	■	■	
	Myrcia fenzliana[2]								■	■	■	■	
	Ormosia arborea[1]	■									■	■	■
	Parkia platycephala[1]									■	■	■	
	Salacia crassifolia[1]	■	■	■	■	■						■	■
	Stryphnodendron adstringens[1]						■	■	■				

[1]Ribeiro *et al.* 2018; [2]Reflora - Virtual Herbarium

Reference:

Ribeiro JF, Peres MK, Santos DS, Sampaio AB, Ogata RS, Souza RM, Oliveira MC, Durigan G, Silva Júnior MC, Sousa FS, Nehme L, Bredt A, Bringel Júnior JBA, Walter BMT. 2018. Época de coleta de frutos e sementes nativas para recomposição ambiental no bioma Cerrado [Time of collection of native fruits and seeds for environmental restoration in the Cerrado biome]. Documentos 374, Embrapa Cerrados. 72 p.
